# Molecular Phenotyping of White Striping and Wooden Breast Myopathies in Chicken

**DOI:** 10.3389/fphys.2020.00633

**Published:** 2020-06-24

**Authors:** Christophe Praud, Justine Jimenez, Eva Pampouille, Nathalie Couroussé, Estelle Godet, Elisabeth Le Bihan-Duval, Cecile Berri

**Affiliations:** ^1^INRAE, Université de Tours, UMR BOA, Nouzilly, France; ^2^Institut Technique de l’Aviculture, Paris, France

**Keywords:** White Striping, Wooden Breast, mitochondria, muscle remodeling, molecular phenotype

## Abstract

The White Striping (WS) and Wooden Breast (WB) defects are two myopathic syndromes whose occurrence has recently increased in modern fast-growing broilers. The impact of these defects on the quality of breast meat is very important, as they greatly affect its visual aspect, nutritional value, and processing yields. The research conducted to date has improved our knowledge of the biological processes involved in their occurrence, but no solution has been identified so far to significantly reduce their incidence without affecting growing performance of broilers. This study aims to follow the evolution of molecular phenotypes in relation to both fast-growing rate and the occurrence of defects in order to identify potential biomarkers for diagnostic purposes, but also to improve our understanding of physiological dysregulation involved in the occurrence of WS and WB. This has been achieved through enzymatic, histological, and transcriptional approaches by considering breast muscles from a slow- and a fast-growing line, affected or not by WS and WB. Fast-growing muscles produced more reactive oxygen species (ROS) than slow-growing ones, independently of WS and WB occurrence. Within fast-growing muscles, despite higher mitochondria density, muscles affected by WS or WB defects did not show higher cytochrome oxidase activity (COX) activity, suggesting altered mitochondrial function. Among the markers related to muscle remodeling and regeneration, immunohistochemical staining of FN1, NCAM, and MYH15 was higher in fast- compared to slow-growing muscles, and their amount also increased linearly with the presence and severity of WS and WB defects, making them potential biomarkers to assess accurately their presence and severity. Thanks to an innovative histological technique based on fluorescence intensity measurement, they can be rapidly quantified to estimate the injuries induced in case of WS and WB. The muscular expression of several other genes correlates also positively to the presence and severity of the defects like *TGFB1* and *CTGF*, both involved in the development of connective tissue, or *Twist1*, known as an inhibitor of myogenesis. Finally, our results suggested that a balance between *TGFB1* and *PPARG* would be essential for fibrosis or adiposis induction and therefore for determining WS and WB phenotypes.

## Introduction

In a context of global population and consumption increase, the world poultry meat supplies have increased from 15 million tons in 1970 to 122.3 million tons in 2017 (FAO, 2019). Chicken alone accounts for 90% of world poultry production. The relative increase in chicken meat consumption is explained by several reasons. It is a coveted source of animal protein, since it is quick and inexpensive to produce. It is not affected by religious prohibitions, and therefore, its consumption is growing in most countries. Its nutritional qualities are of interest, particularly that of the breast meat, which is low in calories, low in fat, and high in protein. Like pork, chicken meat is widely used for processing, although it is also largely consumed as cuts. The main part of poultry meat comes from the production of fast-growing strains whose breast meat yield is high, generally ranging from 20 to 25% depending on age and weight at slaughter (Baéza et al., 2012). These chicken lines are the result of intense genetic selection, mainly based on improving feed efficiency and animal growth, but also increasing their breast meat yields. Less than 10 years ago, the poultry industry had seen the emergence of new quality defects whose incidence has been growing since. Among these, two are predominant: the White Striping (WS) and the Wooden Breast (WB), which are both similar to myopathies.

The WS defect corresponds to the appearance of white striations parallel to the muscle fiber axis. This is mainly observed on the breast *pectoralis major* (PM) muscle and less frequently on other thigh muscles. The WS condition has been first reported in 2012 by Kuttappan et al. (2012b), who proposed a three-point scale classification based on the macroscopic evaluation of the frequency and thickness of white striations present at the surface of the PM muscle. The normal WS0 class regroups muscles with no apparent white striations; the moderate WS1 class, muscles in which white striations are easily recognizable and the severe WS2 class, muscles in which very noticeable and large white striations are observed. The prevalence of WS defect and its progression have been different according countries. The first prevalence estimations were made by American (Kuttappan et al., 2013a), Italian (Petracci et al., 2013), and Brazilian (Ferreira et al., 2014) research teams, in countries known for their very high chicken production performance. According to these studies, the incidence ranged from 10 to 56%. However, this prevalence is likely underestimated, since Trocino et al. (2015) reported a huge difference between the percentage of normal muscles within a broiler population classified by visual observation (18.7%) and that based on microscopic observation of muscle cross-sections (3%). At the microscopic level, muscles affected by WS are characterized by an increase in adipose tissue deposition, fibrosis, inflammatory infiltrates, increased number of internalized nuclei (Kuttappan et al., 2013c), fiber necrosis, and fiber size variability (Mazzoni et al., 2015). The protein content significantly decreases, while the water, collagen, and lipid contents increase between normal and severely affected muscles (Kuttappan et al., 2013b, c; Petracci et al., 2014). Linked with these changes, alteration of protein turnover toward more protein degradation and fat deposition have been reported in WS muscles (Vignale et al., 2017). Evidence of alterations of glucose metabolism, calcium signaling, hypoxia, cell death, and muscle remodeling have been also recently described (Boerboom et al., 2018; Marchesi et al., 2019), as well as the identification of several QTL and candidate genes involved in muscle metabolism, remodeling, or causal in human myopathies (Pampouille et al., 2018).

The WB defect corresponds to a hard and out-bulging muscle condition that principally affects the breast PM muscle. Even though there is no evidence of a common etiology, the WB condition is often associated with the WS one. Although the prevalence of the WB defect has never been precisely described, it seems to be much less than that of WS. WB mainly concerns the production of heavy broilers primarily used for processing. A Finnish team (Sihvo et al., 2013) first described this condition. Macroscopically, the PM muscle appears hard, curved, viscous, and pale. Histologically, WB muscles are characterized by several cellular abnormalities, such as fiber splitting and necrosis, which are associated with excessive adipose tissue deposition, accumulation of interstitial connective tissue and collagen, and inflammatory cells infiltrates (Sihvo et al., 2013; Velleman and Clark, 2015; de Brot et al., 2016; Soglia et al., 2016). There is also strong evidence of muscle cell regeneration associated with increased expression of several myogenic regulatory factors (Clark and Velleman, 2016). High-throughput metabolomic or transcriptomic approaches have also revealed a great number of dysregulated molecular pathways involved in the response to oxidative stress, altered glucose utilization and lipid metabolism, muscle degeneration and repair, inflammatory responses, and extracellular matrix (ECM) remodeling (Abasht et al., 2016, 2019; Kuttappan et al., 2017; Papah et al., 2018; Pampouille et al., 2019). In addition, the WB condition was recently associated with muscle weakness (Norring et al., 2018).

The occurrence of these breast muscle myopathies clearly represents a limit to the sustainability of chicken meat production because of its important economic consequences and unfavorable impact on consumer acceptability. Since the emergence of these defects, numerous research studies have been carried out in order to understand their etiology, but also to propose nutritional and breeding strategies to limit their incidence. There is a consensus today, which links the appearance of these defects to the efficiency and rapid growth of animals and the increase in their breast meat yield (Kuttappan et al., 2012a, 2013b; Kawasaki et al., 2018), and so far, no genetic or rearing solution has made it possible to reduce drastically the occurrence of these defects without affecting growth performance of animals. Another limitation to the search for solutions to reduce the incidence of muscle myopathies in chickens is the lack of quantitative methods to assess the presence and severity of defects. Indeed, most studies are based on visual or palpation estimation of meat defects, even though recent studies have tested the use of spectral methods to detect defects at slaughterhouse (Traffano-Schiffo et al., 2017; Wold et al., 2017). The present study aims to propose histological and molecular tools allowing for precise quantification of the different lesions present in muscles affected by WS or WB. This will ultimately allow progress in understanding the etiology of these defects but also by refining the diagnosis of injuries accelerate the development of non-invasive prediction tools at the service of breeders and producers.

## Materials and Methods

### Animals and Muscles

The muscles used in the present study are issued of a trial already described in Pampouille et al. (2019). Briefly, PM muscles were sampled on 42-day-old chickens originating from two chicken lines: an experimental slow-growing (SG) line showing no lesions related to WS and WB defects (*n* = 8) and a commercial fast-growing (FG) one, in which both WS and WB defects were observed at different degrees (*n* = 32). Within the fast-growing line, WS and WB defects were qualified using a three-point scale for both: 0 in case of absence of defect, 1 when the defect was moderate, and 2 when it was severe. Then, four groups of eight animals each were constituted corresponding to the FG-C muscles that exhibited no defect (WS0 and WB0), the FG-WS muscles that were only affected by a severe WS condition (WS2 and WB0), the FG-WB muscles only affected by WB (balanced between WB1 and WB2 and WS0), and the FG-WSWB muscles severely affected by both defects (WS2 and WB2). Two samples by animal were collected 15 min after slaughter, parallel to the axis of the muscle fibers on the antero-superior part of the PM muscle. The sample dedicated to histology was snap-frozen in isopentane cooled in liquid nitrogen; the other, dedicated to molecular biology, was directly frozen in liquid nitrogen. All muscle samples were stored at −80°C before use.

### Histology and Imaging Procedures

10-μm-thick cross-sections were performed using a Leica CM3050 S cryostat (Leica, Nanterre, France) and deposited on Superfrost plus slides (Thermo Scientific, Montigny-le-bretonneux, France). All 40 muscles were analyzed on a total of eight slides, each of them containing one muscle of each group, representing five muscles by slide that were randomly dropped based on chicken identification number. All cross-sections were stored at −80°C before staining or labeling.

Several stainings were performed, such as hematoxylin and eosin (HE), Gomori trichrome modified by Engel and Cunningham (TG), and reduced nicotinamide adenine dinucleotide-tetrazolium reductase (NADH-TR), following classical methods (Dubowitz et al., 2013) in order to assess the morphological and histochemical properties of the muscle cross-sections.

Regarding immunolabeling, five primary antibodies were obtained from the Developmental Studies Hybridoma Bank, created by the NICHD of the NIH and maintained at The University of Iowa, Department of Biology, Iowa City, IA. They were developed by Douglas M. Fambrough, for the anti-fibronectin (FN1, B3/D6) and the anti-Neural Cell Adhesion Molecule (NCAM, 5e) antibodies; by Everett Bandman, for the anti-ventricular myosin heavy chain 15 (MYH15, HV11) antibody; and by Frank E. Stockdale, for the slow developmental myosin heavy chain 6 (MYH6, S46) and the slow myosin heavy chain 7B (MYH7B, S58) antibodies. Secondary antibodies obtained from Southern Biotech (Birmingham, AL, United States) were Goat anti-mouse IgG H + L biotinylated, Goat anti-mouse IgG1 biotinylated, Goat anti-mouse IgA biotinylated, or Goat anti-mouse IgG1 conjugated with Texas Red. Streptavidin-Cy2 conjugated was obtained from Southern Biotech and streptavidin-Alexa Fluor^®^ 680 (A680), conjugated from Life Technologies (Thermo Scientific, Montigny-le-Bretonneux, France). All primary and secondary antibodies were diluted in goat serum diluted in PBS (Sigma, Saint-Quentin-Fallavier, France) at 1% v/v. Streptavidin were diluted in PBS. All washing steps (3 × 5 min) were performed in PBS.

Muscle cross-sections were first blocked for 30 min in goat serum (Sigma, Saint-Quentin-Fallavier, France) diluted in PBS at 10% v/v. Then, primary antibodies were incubated 2 h at 1/30 for anti-MYH15, anti-MYH6, and anti-MYH7B; and 1/100 for anti-NCAM and anti-FN1. After washing, goat anti-mouse secondary antibodies were incubated for 1 h: IgG1, biotinylated 1/500 for NCAM and MYH15; IgG H + L, biotinylated 1/1,000 for FN1; IgA, biotinylated 1/500 for anti-MYH7B; and IgG1 Texas Red, conjugated for anti-MYH6. When using biotinylated secondary antibodies, sections were then incubated after washing with streptavidin conjugated to either Cy2 or A680.

To quantify adiposis, muscle sections were incubated with Bodipy 493 (4,4′-Difluoro-1,3,5,7,8-Pentamethyl-4-Bora-3a, 4a-Diaza-s-Indacene, 1/3,000, Sigma, Saint-Quentin-Fallavier, France) that is a fluorescent probe staining neutral lipids.

Slides were dehydrated and mounted with Canada balsam (HE and TG) or Moviol (Sigma, Saint-Quentin-Fallavier, France) (NADH-TR and immunohistological labelings), then stored at 4°C protected from light before imaging.

For Bodipy 493 imaging, six images selected randomly for each sample (at 10 × magnification) were acquired using a Leica MC170 color camera on a Leica DMRB epifluorescence microscope (Leica Microsystems SAS, Nanterre, France). The mean area occupied by the fluorescence signal (Bodipy 493 and Cy2) as the percentage on the total area observed was then calculated using Image J 1.44p software (NIH, United States). For all other labeling procedures using A680, slides were scanned using an infrared Odyssey CLX imager (LICOR Biosciences, Bad Homburg, Germany), and the fluorescence intensity was measured on a region of interest covering at least 90% of the area of each muscle sections.

### Cytochrome Oxidase Activity (COX) and Reactive Oxygen Species (ROS) Assays

Both assays were performed from a single sample of 200 mg of PM muscle that was crushed in phosphate buffer 50 mM (KH_2_PO_4_ 50 mM, K_2_HPO_4_ 50 mM, pH 7.0) with an Ultra-Turrax (IKA, Staufen, Germany) and centrifuged 30 min at 10°C to collect supernatant that was further used for the two assays. For cytochrome oxidase activity (COX), enzymatic reaction was done from 100 μg of proteins in phosphate buffer (KH_2_PO_4_ 50 mM and K_2_HPO_4_ 50 mM, pH 7.0) in the presence of cytochrome 0.1 mM, n-dodecylmaltoside 0.15%, and DTT 5 mM (Sigma, Saint-Quentin-Fallavier, France). The reduction of cytochrome c was measured at 550 nm using a Tecan M200 microplate reader (Tecan, Lyon, France). For reactive oxygen species (ROS) assay, 100 μg of proteins was diluted in a reaction buffer (250 mM sucrose, 50 mM KCl, 25 mM Tris-base, 10 mM K_2_HPO_4_, pH 7.4). The oxidation of the 2,7-dichlorodihydrofluorescein diacetate 5 mM by oxygen reactive species was measured at 528 nm using a Spectramax Gemini EM microplate reader (Molecular Devices, Sunnyvale, CA, United States).

### RNA Extraction and RT-qPCR Analysis

Total RNA was extracted from 100 mg of PM muscle with a commercial kit (RNA Now, Ozyme, Saint-Cyr-l’École, France) as described previously (Pampouille et al., 2019). Reverse transcription was done with 10 micrograms of RNA using SuperScript II reverse transcriptase (Invitrogen, Montigny-le-Bretonneux, France), random primers (Promega, Charbonnières-les-Bains, France), and RNAse Out (Invitrogen, Montigny-le-Bretonneux, France) according to manufacturer recommendations. Gene expression quantification was done by qPCR using a Light Cycler 480 (Roche, Meylan, France). Primers were purchased from Eurogentec (Le Tremblay, France) and their sequence are presented in Table 1. PCR conditions were as follow: 5 min of pre-incubation at 95°C, then 45 amplification cycles of 10 s at 95°C, 20 s at 60°C, and 10 s at 72°C. *18S ribosomal RNA* was used as housekeeping gene, and its expression was invariant between groups. The calculation of absolute mRNA levels was based on the PCR efficiency (E) and the threshold cycle (CT) deviation of an unknown cDNA versus the control cDNA according to the equation proposed by Pfaffl (2001): absolute mRNA level of a target gene = (*E*_*target*_)^Δ CTtarget(control–sample)^. To account for variations due to mRNA extraction and reverse-transcription reaction, absolute mRNA levels of targeted genes were corrected for 18S rRNA levels to give a relative mRNA level.

**TABLE 1:** Accession numbers and sequences of the forward and reverse primers used to quantify gene^1^ expression by RT-qPCR.

Gene	Accession number	Forward sequence	Reverse sequence
*18S*	XR_003078044.1	TCCAGCTAGGAATAATGGAATAGGA	CCGGCCGTCCCTCTTAAT
*ADIPOQ*	NM_206991.1	GCCAGGTCTACAAGGTGTCA	CCATGTGTCCTGGAAATCCT
*ANKRD1*	NM_204405.1	GCACAATCATCTGGACACTGG	CACTGAACTGCTGCTCGGTTA
*ANKRD46*	XM_423967.6	GCTAGAAGTACTTACAGCCCTCT	AACCTCTGGCTTAAGAAATGAGA
*ANTXR1*	XM_425758.6	TTGGCGGCATCAAGAGAATG	GTACCACTTCCGAGGAGAGG
*CD36*	NM_001030731	CTGTTTCTCTTTGTGGCCTTTG	CGTGAGAGAAGCTGTATGGAGG
*CTGF*	NM_204274.1	TGAGTGGAGTGCTTGCTCCAAGA	GCAGCCAGACAGCTCAAACTTGAT
*DAG1*	NM_001097540.1	TCCGAAGCTGGGAAGGAGTCTTTG	CGTGGTCACCGAGATGTAGTGGA
*DYSF*	XM_025149877.1	GGTCGGGATGAGCCAAACAT	GTTCGGGAAGGCGTAGATGA
*ENPP2*	NM_001198662.1	ACTGAACGAAATGGAGTCAATGT	TGGTCCATCACACTTGTCGG
*FABP4*	NM_204290.1	CAAGCTGGGTGAAGAGTTTGATGAG	AGCAGGTTCCCATCCACCACTTTT
*FASN*	NM_205155.2	TCTCGATCTGGCATACGAACTG	CAACTGGTCCGAGCTTCAAAG
*FBN1*	XM_015291934.1	AATGCTTCCCTGGCCTTGCAGTA	TGTGCTGGACAGGGCTGACAA
*MTX3*	NM_001079483.1	GCATTTGAAACAGCTTACCAACC	GCGTTGTTATCTTCCGAGGG
*MYH13*	XM_025141815.1	GGAATGACAACTCCTCACGCT	GCTAGCTGGAAAGTCACTCTGG
*MYF5*	NM_001030363.1	ACTCCCCAAAGTGGAGATCCTG	CAGTCCGCCATCACATCG
*MYOD1*	NM_002478.5	GGAATCACCAAATGACCCAAAG	TCACTCAGGTTTCCTCCTTCCT
*MYOG*	NM_204184.1	CGGAGGCTGAAGAAGGTGAAC	AGGCGCTCGATGTACTGGAT
*PCNA*	NM_204170.1	CATGGGCGTCAACCTAAAC	TCCACATCGAGGTCCATAAG
*PDE3B*	NM_001031182.1	CACCATCTCAGCGAAAATCACA	TCTCCTAGCTGCCGCTCATCTT
*PDGFRA*	NM_204749.2	CTTGGAGCTGTTACTCGGGAACTGA	ACCTTGGCTTCTGTTTCCAGATGA
*PITX2*	NM_205010.1	GTCTGGACCAACCTCACCG	TGCCCAGTTGTTGTACGAGT
*PLIN2*	NM_001031420.1	GCCTACATCACCACGAAGGATAACC	CAACCTTGTCAGTGGGTTGATTCAG
*PNPLA7*	NM_001130742.1	TTTCACCATCAAGGCCAATCG	GTCCAGCCTCAACTTCCATCCA
*PPARG*	NM_001001460.1	CACTGCAGGAACAGAACAAAGAA	TCCACAGAGCGAAACTGACATC
*SGCB*	NM_001031155.1	TGTAGAGCGCAGGAACGTCAAT	AATCACCGCCCAGATAACGAG
*TGFB1*	NM_001318456.1	ACCCGATGAGTATTGGGCCAAAGA	GCGGGACACGTTGAACACGAA
*TUBB4B*	NM_001080860.2	CAGACCGGATTATGAACACCTT	CACCGTATGTTGGCGTAGTT
*TWIST1*	NM_204739.1	CGCAGTCCCTGAACGAAGCTTT	CACACCGAGAAGGCGTAGCTGA

*^1^Primers used to measure the expression of genes not listed in this table are described in Pampouille et al. (2019).*

### Statistical Analysis

Data are presented as mean ± SEM and analyzed using Statview 5.0 Software (SAS Institute, Inc.). One-way ANOVA was performed in case of normally distributed and homoscedastic variance. Statistical significance (*P* ≤ 0.05) was determined using the *post hoc* Fisher test. Data that did not meet assumptions of normality and homoscedasticity were analyzed and statistical significance (*P* ≤ 0.05) was determined using Kruskal–Wallis and Mann–Whitney non-parametric tests, respectively. Pearson’s correlation coefficients were calculated between the signal quantification obtained by microscopy and scanner technology and between values of mRNA expression of each gene studied.

## Results

### Histological Description

**FIGURE 1:**
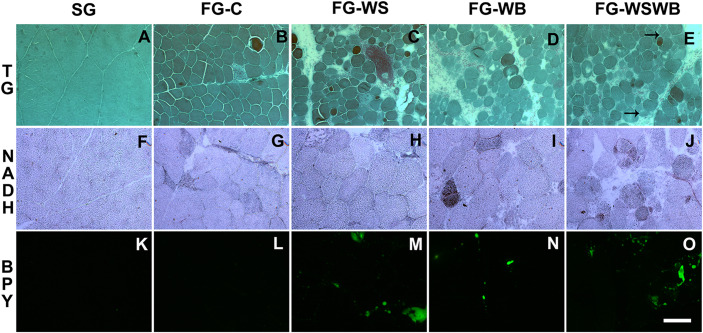
Histological characterization of *pectoralis major* muscle using Gomori’s Trichrome (TG, **A–E**), NADH-TR (NADH, **F–J**) and BODIPY493 (BPY, **K–O**) staining. Stainings were performed on *pectoralis major* muscles from a slow-growing genotype **(A,F,K)** and a fast-growing genotype macroscopically free of defects **(B,G,L)** or affected by White Striping **(C,H,M)**, Wooden Breast **(D,I,N)**, or both White Striping and Wooden Breast **(E,J,O)**. Bar represents 98 μm **(A–E)** and 200 μm **(F–O)**. Black arrows indicate regenerative fibers consecutive to segmental fiber necrosis.

Muscle characterization was first performed on HE- and TG-stained cross-sections in order to have an overview of muscle structure and the kind of damages according to the group. SG muscles are made of polygonal fibers containing both internalized and peripheral nuclei. Muscle fibers are surrounded by normal thick endomysium and perimysium without any detectable damage or additional deposit of intermuscular adipose tissue (adiposis) (Figure 1A). By comparison, FG-C muscles contain fibers of more variable sizes and exhibit a slightly extended endomysium and perimysium, first signs of adiposis, some necrotic and regenerating fibers, and rare hypercontracted fibers and vacuoles within muscle fibers (Figure 1B). The presence of internalized nuclei was observed in almost all fibers of FG muscles, whether WS or WB defects were present or not. Muscles only affected by WS (FG-WS) exhibit round fibers of variable size and greater extension of endomysium and perimysium compared to FG-C. These muscles show adiposis essentially in the perimysium, some necrotic and regenerated fibers, and some hypercontracted fibers and rare inflammatory cells foci (Figure 1C). In muscles only affected by WB (FG-WB), the number of round fibers increases, the extension of endomysium and perimysium is more pronounced, while adiposis decreases compared to FG-WS muscles. Events of fiber necrosis, regeneration, hypercontracted fibers, and vacuoles also increase (Figure 1D). Finally, muscles affected by both defects (FG-WSWB) look like FG-WB muscles, with an increased adiposis (Figure 1E). In muscles affected by WB (FG-WB and FG-WSWB) but not in those affected by WS, some fibers looked segmented.

To explore the metabolic status of muscle fibers, an NADH-TR staining was performed. All SG muscle fibers were weakly stained (Figure 1F). Moreover, they did not present any immunoreactivity, with MYH6 and MYH7B staining, respectively, a slow developmental and a slow myosin heavy chain (data not shown). Some fibers showed a higher staining on NADH-TR in FG-C (Figure 1G) and in FG-WS (Figure 1H) muscles that was usually associated with an immunoreactivity with MYH6, but not with MYH7B (data not shown). The proportion of high NADH-TR stained fibers increased in FG-WB (Figure 1I) and in FG-WSWB (Figure 1J) muscles. This was associated with an increase in MYH6 but not MYH7B positive fibers (data not shown).

**FIGURE 2:**
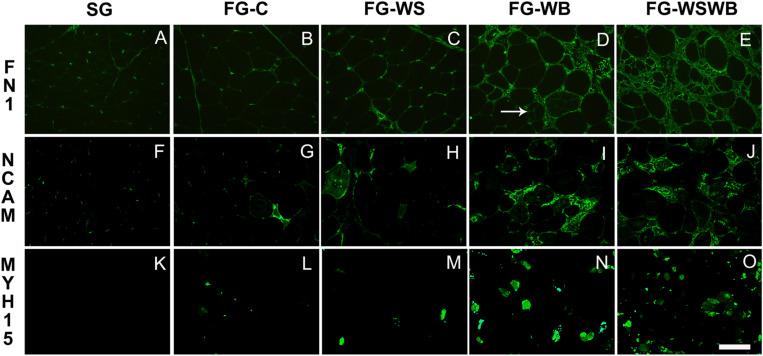
Immunoreactivity for fibronectin (FN1, **A–E**), NCAM **(F–J)** and MYH15 **(K–O)**. It was assessed on *pectoralis major* muscles from a slow-growing genotype **(A,F,K)** and a fast-growing genotype macroscopically free of defects **(B,G,L)** or affected by White Striping **(C,H,M)**, Wooden Breast **(D,I,N)**, or both White Striping and Wooden Breast **(E,J,O)**. Bar represents 200 μm **(A–J)** and 98 μm **(K–O)**. White arrow indicates regenerative fiber consecutive to segmental fiber necrosis.

To improve description and quantify the different features of WS and WB conditions, we applied several stainings using either specific antibodies against fibronectin, MYH15 and NCAM, or the fluorescent primer BODIPY493 (Figures 1, 2). In SG muscles, adiposis was absent or very rare (Figure 1K), fibronectin was only present around capillaries and in perimysium (Figure 2A), and NCAM was expressed in mononuclear and endothelial cells (Figure 2F). SG muscles did not express MYH15 (Figure 2K). Comparatively, fibronectin was also detected in the endomysium (Figure 2B), NCAM in the cytoplasm of few small fibers (Figure 2G), and MYH15 was detected in few small fibers and mononuclear cells (Figure 2L) in FG-C muscles. Within affected muscles, although small adiposis areas were present in FG-WB (Figure 1N), their size and number greatly increased in FG-WS (Figure 1M) and in FG-WSWB (Figure 1O). Fibronectin labeling extended in both the endomysium and perimysium in FG-WS (Figure 2C), but especially in FG-WB (Figure 2D) and FG-WSWB (Figure 2E). It was also present around small fibers formed after segmental necrosis in FG-WB muscles (Figure 2D). Finally, the number of fibers expressing NCAM (Figure 2I) and MYH15 (Figure 2N) strongly increased in FG-WB muscles, and even more in FG-WSWB, in which the size of the MYH15 marked fibers was also larger (Figures 2J,O).

### Quantification of Histological Phenotypes

**FIGURE 3:**
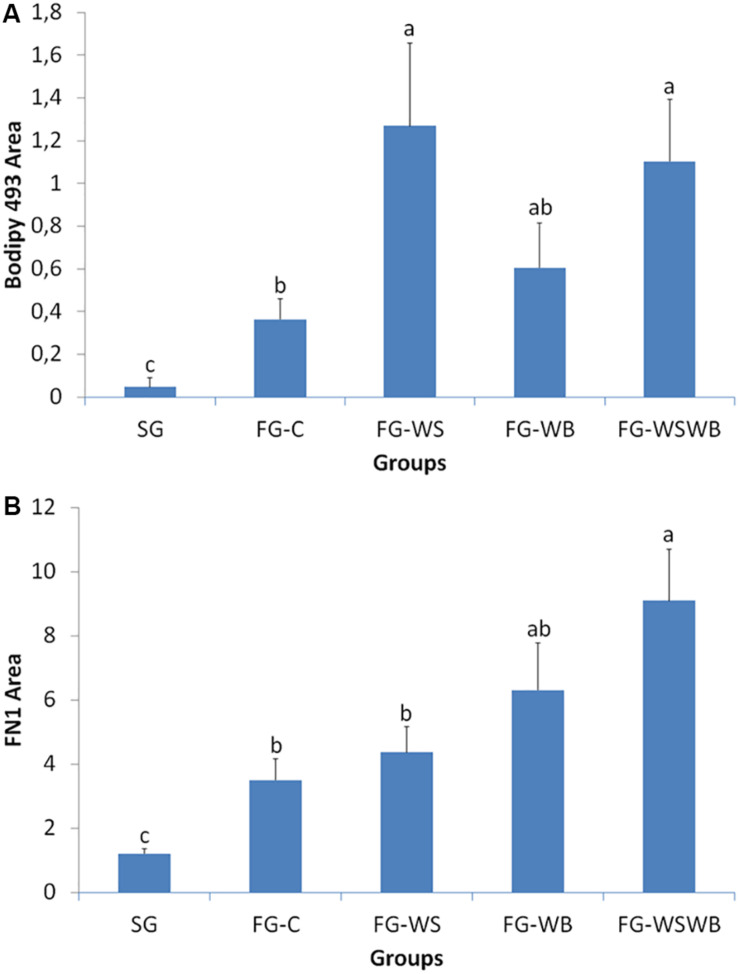
Quantification of the percentage area labeled by Bodipy493 **(A)** and fibronectin (FN1, **B**) on *pectoralis major* muscle cross-sections using classical microscopy. Labeled areas were assessed in a slow-growing genotype (SG) and a fast-growing genotype macroscopically free of defects (FG-C) or affected by White Striping (FG-WS), Wooden Breast (FG-WB), or both White Striping and Wooden Breast (FG-WSWB). Bodipy-493-labeled area increased mostly in muscles affected by White Striping (FG-WS and FG-WSWB). FN1-labeled area increased linearly with the occurrence and severity of both White Striping and Wooden Breast defects. For each labeling, different letters indicate significant differences between muscle groups (*P* ≤ 0.05).

The quantification of Bodipy 493 and FN1 labeling was performed on images classically obtained by microscopy and analyzed with the ImageJ software to measure percentage of fluorescent area. The area percentage labeled by Bodipy493 was largely higher in FG compared to SG muscles (Figure 3A). It was about 10 times higher in FG-C and around 30 times higher in muscles affected by WS (FG-WS and FG-WSWB), FG-WB muscles being intermediate between muscles affected by WS and control ones. The percentage area occupied by FN1 was lower in SG compared to FG muscles (Figure 3B). Among FG muscles, the highest value was observed in muscles affected by both WS and WB defects (FG-WSWB), FG-C and FG-WS muscles exhibiting the lowest values, and FG-WB being intermediate.

**FIGURE 4:**
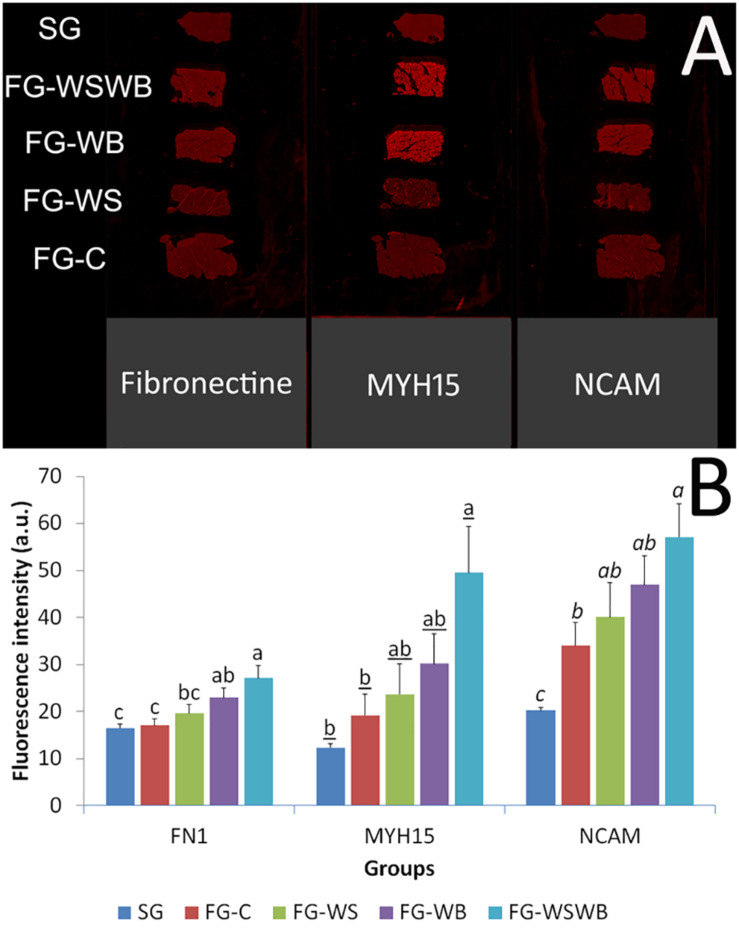
Quantification of the fluorescence intensity following fibronectin, myosin heavy chain 15 (MYH15), and Neural Cell Adhesion Molecule (NCAM) immunostaining using an infrared scanner Odyssey CLX. Labeling were performed on *pectoralis major* muscles from a slow-growing genotype (SG) and a fast-growing genotype macroscopically free of defects (FG-C) or affected by White Striping (FG-WS), Wooden Breast (FG-WB), or both White Striping and Wooden Breast (FG-WSWB) **(A)**. The fluorescence intensity increased linearly with the occurrence and severity of both White Striping and Wooden Breast defects for Fibronectin, MYH15, and NCAM **(B)**. For each marker, different letters indicate significant differences between muscle groups (*P* ≤ 0.05).

To quantify NCAM and MYH15 signals, we developed a tissue section imaging protocol based on immunohistochemistry (IHC) fluorescent signal measurement using an Odyssey CLX imager. The interest in developing such a method is time saving for image acquisition and the possibility of analyzing the whole muscle cross-section (Figure 4A). Unfortunately, the implementation of this technique has not yet been possible for the labeling of adipose tissue by the Bodipy 493, as this probe is not adapted to the acquisition wavelength of our scanner. Before deploying this method, we needed to validate that the quantification of the fluorescent signal obtained using the scanner correlated with area calculation performed on images obtained under the microscope. Thus, the fluorescent signals of 10 samples from the different groups (corresponding to two slides) were analyzed using both a classical epifluorescent microscope and scanner technology. Pearson correlations between the signal quantification obtained by the two methodologies were very high: 0.969, 0.873, and 0.977 (*P* ≤ 0.001) for NCAM, fibronectin, and MYH15 signals, respectively.

The fluorescent signal for the three markers studied obtained using the scanner was very low for SG muscles (Figure 4A), as also observed with the epifluorescence microscope (see Figure 1 for fibronectin). The gross observation of images acquired using the scanner technology revealed that whatever the labeling the intensity of fluorescence increased with the increase of damages observed, especially in FG-WB and FG-WSWB (Figure 4A). Fibronectin labeling allowed observing distinctly perimysium in FG-C and FG-WS, but not in SG muscles, where it was poorly developed, and in FG-WB and FG-WSWB muscles, where endomysium was the most extended, compared to other groups. Likely because of their localization in mononuclear and muscle fiber cells, increased fluorescent signal due to alterations revealed by MYH15 and NCAM labeling in muscles affected by WB was more evident than in fibronectin. FN1 signal quantification showed a similar pattern to that obtained by microscopy, except for an absence of significant difference between SG and FG-C and FG-WS muscles (Figure 4B). Like for fibronectin, NCAM and MYH15 signals progressively increased with the intensity of defect, SG and/or FG-C muscles showing the lowest values and FG-WSWB the highest (Figure 4C). For all markers, FG-WS and FG-WB muscles exhibited intermediate values (Figures 4B,C). The Pearson correlation coefficient between MYH15 and NCAM signal was very high: 0.915 (*P* ≤ 0.001).

### COX Activity and ROS Assay

**FIGURE 5:**
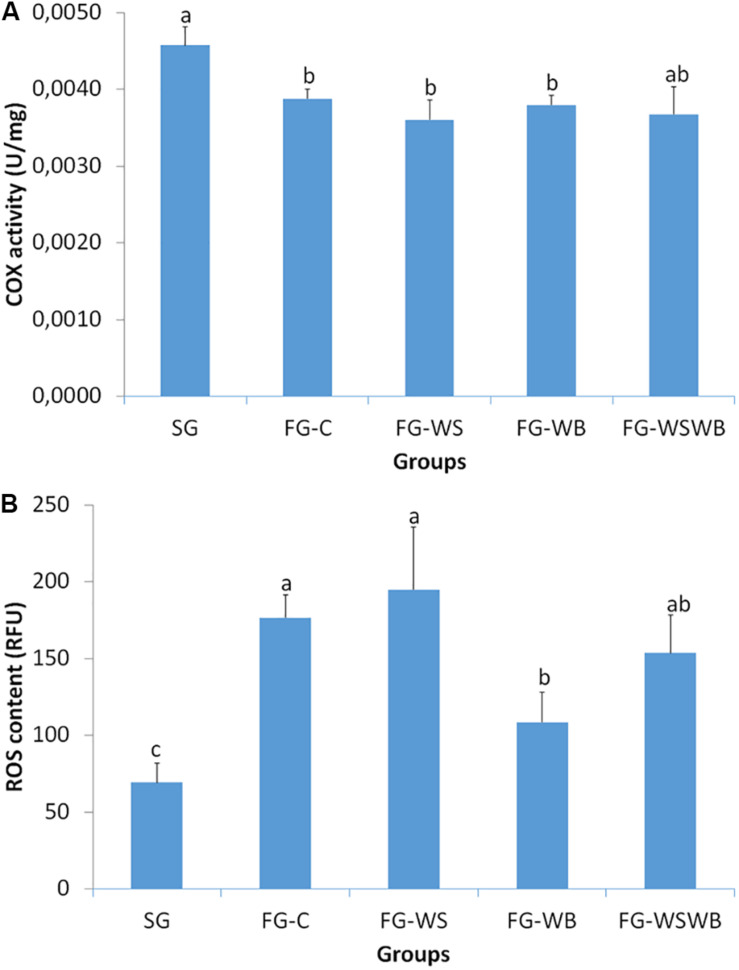
Quantification of the cytochrome oxidase (COX) activity and the production of reactive oxygen species (ROS). These parameters were assessed in *pectoralis major* muscles from a slow-growing genotype (SG) and a fast-growing genotype macroscopically free of defects (FG-C), or affected by White Striping (FG-WS), Wooden Breast (FG-WB), or both White Striping and Wooden Breast (FG-WSWB). Fast-growing muscles exhibited lower COX activity than slow-growing muscles, independently of the defect occurrence and severity **(A)**. ROS content was higher in fast- than in slow-growing muscles, especially those affected by White Striping **(B)**. For each parameter, different letters indicate significant differences between groups (*P* ≤ 0.05).

We assessed mitochondrial activity of muscles by measuring the COX enzymatic activity. COX activity was significantly higher in SG than in FG muscles, except for the FG-WSWB group, whose variability of activity between individuals was more important than for other groups (Figure 5A). By contrast, the production of ROS was lower in SG than in FG muscles, in which the FG-C and FG-WS muscles exhibited the highest values and the FG-WSWB muscles the lowest one (Figure 5B).

### Gene Expression

**FIGURE 6:**
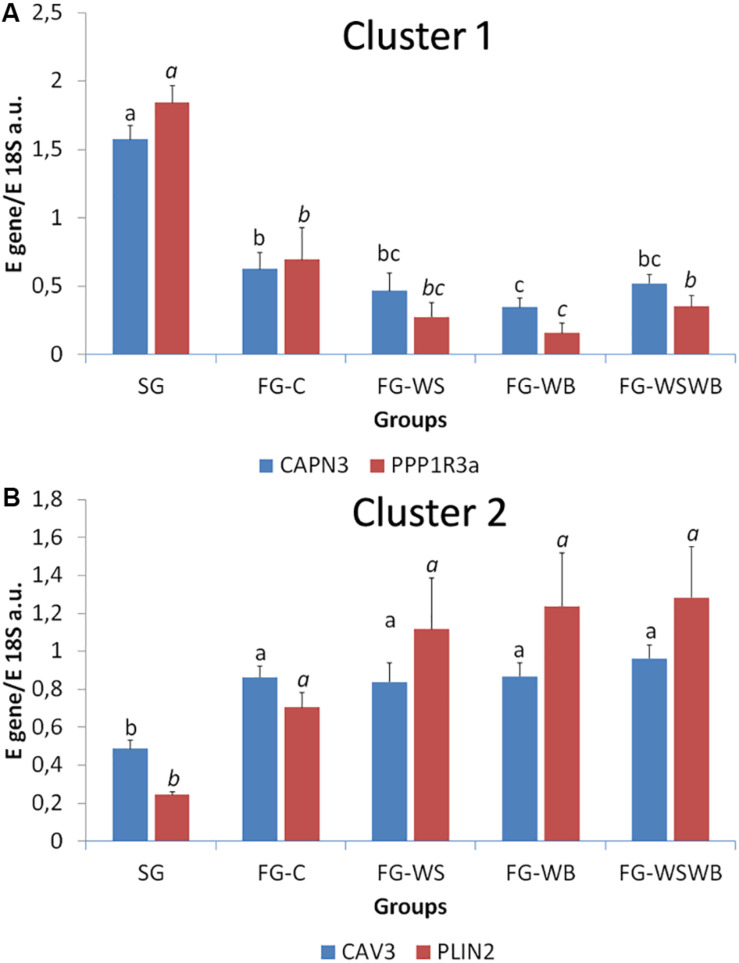
Relative mRNA expression of genes coding for calpain 3 (*CAPN3*), protein phosphatase 1 regulatory subunit 3A (*PPP1R3A*), caveolin 3 (*CAV3*), and perilipin 2 (*PLIN2*). mRNA expression was assessed in *Pectoralis major* muscles from a slow-growing genotype (SG) and a fast-growing genotype macroscopically free of defects (FG-C) or affected by White Striping (FG-WS), Wooden Breast (FG-WB), or both White Striping and Wooden Breast (FG-WSWB). Expression of *CAPN3* and *PPP1R3A* was higher in slow-compared to fast-growing muscles **(A)**. *CAV3* and *PLIN2* expressed more in fast- than in slow-growing muscles **(B)**. Absolute mRNA levels of targeted genes were corrected for *18S ribosomal RNA* levels to give a relative mRNA level. For each gene, different letters indicate significant differences between groups (*P* ≤ 0.05).

**FIGURE 7:**
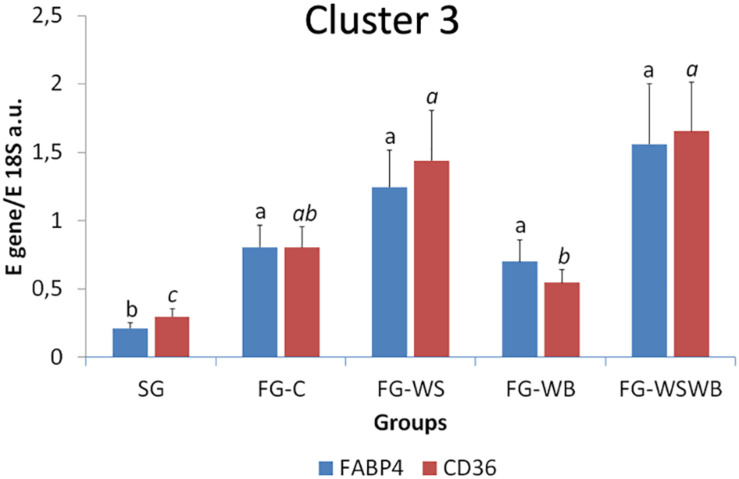
Relative mRNA expression of genes coding for fatty acid binding protein 4 (*FABP4*) and cluster of differentiation 36 (*CD36*). mRNA expressions were assessed in *pectoralis major* muscles from a slow-growing genotype (SG) and a fast-growing genotype macroscopically free of defects (FG-C) or affected by White Striping (FG-WS), Wooden Breast (FG-WB), or both White Striping and Wooden Breast (FG-WSWB). *FABP4* and *CD36* expressed also more in fast- than in slow-growing muscles, especially in muscles affected by White Striping. Absolute mRNA levels of targeted genes were corrected for *18S ribosomal RNA* levels to give a relative mRNA level. For each gene, different letters indicate significant differences between groups (*P* ≤ 0.05).

**FIGURE 8:**
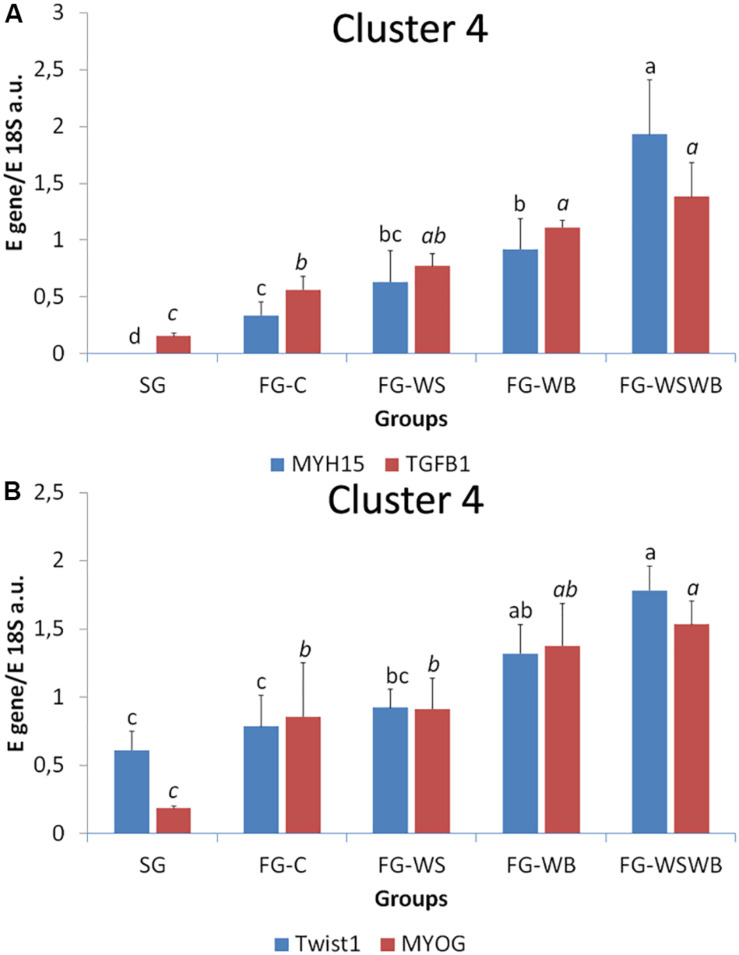
Relative mRNA expression of genes coding for myosin heavy chain 15 (*MYH15*), transforming growth factor beta 1 (*TGFB1*), Twist basic helix-loop-helix transcription factor 1 (*Twist1*), and myogenin (*MYOG*). mRNA expressions were assessed in *pectoralis major* muscles from a slow-growing genotype (SG) and a fast-growing genotype macroscopically free of defects (FG-C) or affected by White Striping (FG-WS), Wooden Breast (FG-WB), or both White Striping and Wooden Breast (FG-WSWB). Expression of *MYH15*, *TGFB1* **(A)**, *Twist1*, and *MYOG* **(B)** increased linearly with the occurrence of White Striping and Wooden Breast defects. Absolute mRNA levels of targeted genes were corrected for *18S ribosomal RNA* levels to give a relative mRNA level. For each gene, different letters indicate significant differences between groups (*P* ≤ 0.05).

**FIGURE 9:**
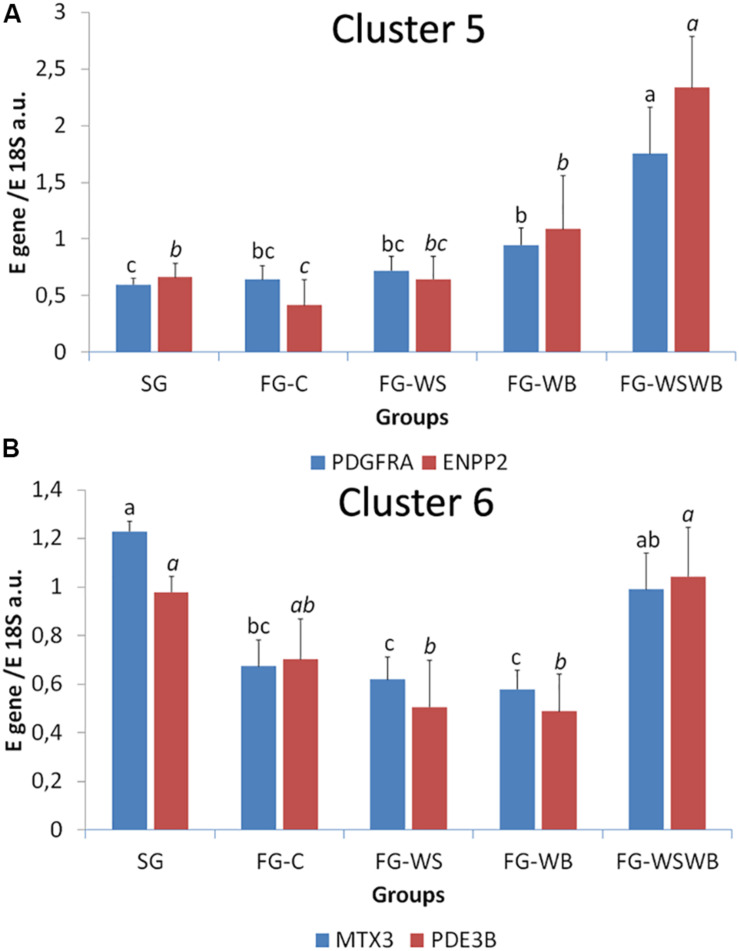
Relative mRNA expression of genes coding for platelet derived growth factor receptor alpha (*PDGFRA*), Ectonucleotide Pyrophosphatase/Phosphodiesterase 2 (*ENPP2*), metaxin 3 (*MTX3*), and phosphodiesterase 3B (*PDE3B*). mRNA expressions were assessed in *pectoralis major* muscles from a slow-growing genotype (SG) and a fast-growing genotype macroscopically free of defects (FG-C) or affected by White Striping (FG-WS), Wooden Breast (FG-WB), or both White Striping and Wooden Breast (FG-WSWB). Expression of *PDGFRA* and *ENPP2* increased specifically in FG-WSWB muscles **(A)**. Expression of *MTX3* and *PDE3B* was high in both SG and FG-WSWB muscles **(B)**. Absolute mRNA levels of targeted genes were corrected for *18S ribosomal RNA* levels to give a relative mRNA level. For each gene, different letters indicate significant differences between groups (*P* ≤ 0.05).

**FIGURE 10:**
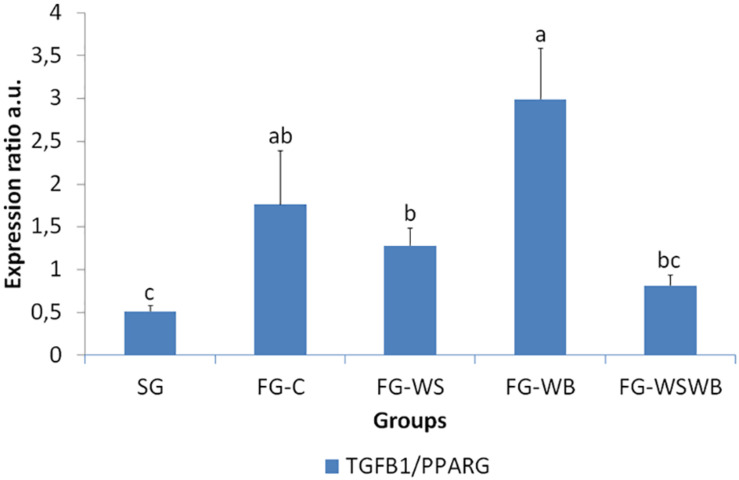
Ratio *TGFB1* on *PPARG* relative mRNA expression. It was assessed in *pectoralis major* from a slow-growing genotype (SG) and a fast-growing genotype macroscopically free of defects (FG-C) or affected by White Striping (FG-WS), Wooden Breast (FG-WB), or both White Striping and Wooden Breast (FG-WSWB). *TGFB1*/*PPARG* ratio was the lowest in SG and the highest in FG-WB muscles, suggesting that a high ratio would favor the development of a fibrotic rather than adipogenic phenotype. Absolute mRNA levels of targeted genes were corrected for *18S ribosomal RNA* levels to give a relative mRNA level. Different letters indicate significant differences between groups (*P* ≤ 0.05).

A set of 39 genes were selected for expression analysis, based on several criteria related to their expressional pattern already described in SG, FG-C, FG-WSWB (Pampouille et al., 2019), their belonging to QTL regions controlling WS or breast muscle yield in chicken (Pampouille et al., 2018), and/or their role in mammalian myopathies or in regeneration or myogenesis processes. Their levels of expression relative to *18S ribosomal RNA* are presented in Table 2 and in Figures 6–9. Pearson’s correlations between the relative mRNA levels of genes differentially expressed in PM muscle are presented in Supplementary File 1. Of the 39 studied genes, 11 were not differential between groups: *DAG1*, *SGCB*, *DYSF*, *ADIPOQ*, *FBN1*, *MYOD1*, *MYF5*, *PITX2*, *ANKRD1*, *LRSAM*, and *PNPLA7* (Table 2). The 28 differential genes could be grouped into six clusters with specific expression profiles. A first cluster (cluster 1) regroups seven genes whose expression is higher in SG than FG muscles. It includes *MYH13*, *MYH1E*, *MYH1F*, *ANKRD46*, *MYOCD* (Table 2), *CAPN3*, and *PPP1R3A* (Figure 6A), despite *MYH1F* being only numerically upregulated (*P* = 0.09) in FG-C compared to SG. In addition to being more expressed in SG muscles, some of them were also less expressed in muscles affected by WB than in fast-growing control ones (*MYH13*, *MYH1E*, *ANKRD46*, *MYOCD*; Table 2). Among genes belonging to cluster 1, the expressions of *MYOCD*, *CAPN3*, *PPP1R3A*, and *MYH1E* were highly positively correlated (Supplementary File 1). The second cluster (cluster 2) regroups genes whose expression was higher in FG than in SG muscles, independently of the presence or severity of the defects. This is the case of *PLIN2* and *CAV3* (Figure 6B), *MYH1B*, and *TUBB4B* (Table 2). A third cluster (cluster 3) brings together two genes, *FABP4* and *CD36*, whose expression was particularly upregulated in muscles affected by WS, even if it is not necessarily different from other FG groups (Figure 7). The Pearson correlation coefficient between the expression of *FABP4* and *CD36* was 0.81 (*P* ≤ 0.001) suggesting common regulation (Supplementary File 1). The fourth cluster (cluster 4) groups genes whose expression is only significantly increased in the presence of WB and even more when WB is associated with WS (compared to FG-C). This is the case of *MYH15* and *TGFB1* (Figure 8A), *TWIST1* and *MYOG* (Figure 8B), and *CTGF* and *FN1* (Table 2). The fifth cluster (cluster 5) regroups genes whose expression is systematically higher in muscles affected by both WS and WB (FG-WSWB) compared to other groups, although differences between other groups sometimes exists. This is the case for *PDGFRA*, *ENPP2* (Figure 9A), *COL6A3*, *FASN*, *PPARG*, and *ANTXR1* (Table 2). Among genes of clusters 4 and 5, *MYH15* appeared to be very positively correlated (R > 0.7) with several genes involved in the processes of fibrosis (*CTGF*, *FN1*, *COL6A3*) and adiposis (*PDGFRA*, *PPARG*, *FASN*) (Supplementary File 1). Finally, a sixth cluster (cluster 6) brings together genes like *MTX3*, *PDE3B* (Figure 9B), and *PCNA* (Table 2) that, despite an expression higher in SG compared to control FG muscles, were also highly expressed in muscles severely affected by both WS and WB (Figure 9). Ratio of expression *TGFB1/PPARG* was calculated to evaluate its potential role in the development of fibrotic or adipogenic phenotypes. It was the lowest in SG and the highest in FG-WB muscles, other groups showing intermediate values (Figure 10).

**TABLE 2:** Effect of muscle group^1^ on the relative^2^ mRNA expression measured in *pectoralis major* muscle.

Gene (cluster)	SG	FG-C	FG-WS	FG-WB	FG-WSWB	*P*
*MYH13 (1)*	2.10 ± 0.45 (a)	0.98 ± 0.25 (b)	0.54 ± 0.13 (bc)	0.30 ± 0.07 (c)	0.29 ± 0.05 (c)	0.005
*MYH1E (1)*	2.49 ± 0.22 (a)	0.66 ± 0.20 (b)	0.29 ± 0.08 (bc)	0.17 ± 0.05 (c)	0.21 ± 0.04 (c)	0.0002
*MYH1F (1)*	1.39 ± 0.24 (a)	0.86 ± 0.15 (ab)	0.60 ± 0.11 (b)	0.47 ± 0.18 (b)	0.56 ± 0.13 (b)	0.02
*ANKRD46 (1)*	713.6 ± 222.7 (a)	5.72 ± 2.86 (b)	1.86 ± 0.45 (bc)	1.36 ± 0.61 (c)	0.86 ± 0.16 (c)	≤0.0001
*MYOCD (1)*	1.87 ± 0.13 (a)	0.77 ± 0.19 (b)	0.53 ± 0.06 (b)	0.34 ± 0.03 (c)	0.55 ± 0.09 (bc)	0.0002
*TUBB4B (2)*	0.57 ± 0.07 (b)	0.85 ± 0.13 (a)	0.93 ± 0.09 (a)	1.15 ± 0.17 (a)	1.03 ± 0.12 (a)	0.01
*MYH1B (2)*	0.05 ± 0.01 (b)	0.96 ± 0.28 (ab)	0.75 ± 0.17 (a)	0.57 ± 0.16 (a)	1.26 ± 0.18 (a)	≤0.0001
*CTGF (4)*	0.11 ± 0.03 (d)	0.30 ± 0.05 (c)	0.61 ± 0.16 (bc)	0.86 ± 0.12 (ab)	1.46 ± 0.27 (a)	≤0.0001
*FN1 (4)*	0.11 ± 0.02 (d)	0.45 ± 0.21 (c)	0.62 ± 0.14 (bc)	1.18 ± 0.25 (ab)	1.74 ± 0.24 (a)	≤0.0001
*FASN (5)*	0.31 ± 0.03 (c)	0.61 ± 0.12 (b)	0.87 ± 0.14 (ab)	0.91 ± 0.11 (ab)	1.41 ± 0.38 (a)	0.0007
*ANTXR1 (5)*	1.00 ± 0.14 (b)	0.70 ± 0.22 (b)	0.73 ± 0.19 (b)	0.85 ± 0.18 (b)	2.04 ± 0.48 (a)	0.04
*PPARG (5)*	0.31 ± 0.03 (c)	0.69 ± 0.23 (bc)	0.70 ± 0.16 (b)	0.60 ± 0.22 (bc)	2.04 ± 0.56 (a)	0.002
*COL6A3 (5)*	0.39 ± 0.05 (c)	0.71 ± 0.18 (bc)	0.82 ± 0.12 (b)	1.05 ± 0.13 (b)	1.53 ± 0.15 (a)	0.0005
*PCNA (6)*	1.19 ± 0.13 (c)	0.78 ± 0.12 (bc)	0.79 ± 0.12 (b)	1.01 ± 0.13 (b)	1.30 ± 0.20 (a)	0.05
*MyoD*	0.84 ± 0.08	0.71 ± 0.09	0.84 ± 0.20	0.82 ± 0.11	0.75 ± 0.11	NS
*MYF5*	1.25 ± 0.19	1.63 ± 0.34	1.08 ± 0.15	1.53 ± 0.41	1.76 ± 0.38	NS
*PITX2*	0.67 ± 0.20	0.82 ± 0.17	1.19 ± 0.33	1.65 ± 0.40	1.06 ± 0.17	NS
*DAG1*	1.05 ± 0.13	0.89 ± 0.13	0.86 ± 0.09	0.80 ± 0.10	1.13 ± 0.40	NS
*PNPLA7*	1.14 ± 0.12	0.81 ± 0.10	0.74 ± 0.06	0.85 ± 0.07	0.91 ± 0.09	NS
*LRSAM*	0.75 ± 0.08	0.78 ± 0.10	0.91 ± 0.10	0.95 ± 0.11	1.07 ± 0.14	NS
*ADIPOQ*	0.84 ± 0.18	0.68 ± 0.10	0.77 ± 0.04	0.62 ± 0.08	0.95 ± 0.27	NS
*FBN1*	1.12 ± 0.26	0.55 ± 0.12	0.66 ± 0.08	0.64 ± 0.09	1.35 ± 0.55	NS
*DYSF*	0.99 ± 0.09	0.86 ± 0.06	0.82 ± 0.06	0.76 ± 0.04	0.88 ± 0.10	NS
*ANKRD1*	0.51 ± 0.11	0.73 ± 0.18	0.68 ± 0.20	0.68 ± 0.17	1.17 ± 0.23	NS

*Data are presented as mean ± SEM. Within a line, different letters indicate significant differences between groups (*P* ≤ 0.05). ^1^SG = slow-growing genotype, FG-C = fast-growing genotype macroscopically free of defects, FG-WS = fast-growing genotype affected by White Striping, FG-WB = fast-growing genotype affected by Wooden Breast, FG-WSWB = fast-growing genotype affected by both White Striping and Wooden Breast. ^2^Absolute mRNA levels of targeted genes were corrected for *18S ribosomal RNA* levels to give a relative mRNA level.*

## Discussion

White Striping (WS) and Wooden Breast (WB) have been widely characterized, especially at the histological level. One of the objectives of the present study was to propose additional innovative histological methodologies to better quantify the different injuries usually associated with these two defects. For that purpose, we used a set of 40 muscles, which were first characterized both macroscopically and microscopically to precisely define the severity of each of the two defects. Among them, eight muscles were from a slow-growing line whose average body weight and breast meat yield at 42 days were much lower than that of the fast-growing line to which it was compared (Pampouille et al., 2019). The aim of including slow-growing muscles was to have control muscles with no lesions typical of WS and WB defects, since all the fast-growing muscles, including normal ones, had them. As already observed (Kuttappan et al., 2013c; Sihvo et al., 2013), we confirmed that muscles affected by WS exhibit adiposis, i.e., an intermuscular adipose tissue deposition, fibrosis, fiber necrosis and regeneration, and some inflammatory infiltrates, and that increased occurrence of fiber necrosis, fibrosis and adipose tissue infiltration are well correlated with the severity of WS (Russo et al., 2015). Our results also supported that damages like fibrosis, hyaline muscle degeneration, and inflammatory cell infiltrate worsen in muscles affected by WB compared to WS, but not necessarily adiposis (Velleman and Clark, 2015; de Brot et al., 2016; Soglia et al., 2016). We also observed an increased number of basophilic fibers, which are known as hypercontracted fibers (Dubowitz et al., 2013), and of hyaline vacuoles in fiber sarcoplasm. Finally, our observations revealed an alteration of the fiber regeneration process in WB muscle as it seemed preferentially performed consecutive to a segmental fiber necrosis (arrows in Figures 1, 2).

### Improving WS and WB Diagnosis Using Histological Phenotypes

Since their appearance less than 10 years ago, most of studies classified WS and WB muscles according to macroscopic visual and/or palpation scores, which are subjective criteria, likely dependent of the person performing them. The limits of these methods were raised by Trocino et al. (2015), and more recently by Pampouille et al. (2019), who revealed discrepancies between muscle categorization made by visual notation or using histological criteria. This last study was the first one to propose quantitative histological phenotype based on fluorescent labeling of adiposis and fibrosis to refine WS and WB diagnosis (Pampouille et al., 2019). In the present study, we proposed other quantitative histological traits able to assess more features of WS and WB conditions, including connective tissue deposition, fiber necrosis, and regeneration, by using antibodies against fibronectin, NCAM, and MYH15. Fibronectin is a component of the ECM surrounding muscle fibers. MYH15 is specific of a chicken ventricular myosin heavy chain that is known to be expressed in developing and regenerating avian skeletal muscles (Camoretti-Mercado et al., 1993). Both fibronectin and MYH15 were chosen because their gene expression was much higher (×8 for *FN1* and ×12 for *MYH15*) in fast-growing muscles affected by both WS and WB than in normal FG muscles (Pampouille et al., 2019). They are also located in QTL regions controlling WS (Pampouille et al., 2018). The neural cell adhesion molecule (NCAM), also called CD56, is a glycoprotein expressed in skeletal muscle regenerative fibers and is considered a good index of muscle regeneration in dystrophin-deficient MDX mouse (Dubois et al., 1994). The present study revealed that fibronectin is clearly more expressed in the most fibrotic muscles and that the area percentage occupied by fibronectin on muscle cross-sections progressively increased from SG to FG-WSWB muscles (SG < FG-C < FG-WS < FG-WB < FG-WSWB; Figures 1, 3). MYH15 labeling showed that it was expressed in regenerating fibers of various size, the fluorescent signal being, however, more intense in the smallest fibers and in some mononuclear cells, which could be myoblasts entering differentiation (Figure 1). This was consistent with the fact that MYH15 is expressed in developing and regenerating muscles (Camoretti-Mercado et al., 1993). As expected, the surface occupied by NCAM labeling greatly increased in muscle showing signs of muscle regeneration, especially those affected by WB (Figure 1). Differences observed between MYH15 and NCAM were likely due to the higher persistence of NCAM expression in more mature fibers than of MYH15, which is consistent with the greatest size of NCAM expressing fibers on immunohistochemistry.

Because the quantification of the fluorescent signal by conventional microscopy is long and not necessarily compatible with the study of large numbers, we developed a rapid method to quantify fluorescence intensity, based on the use of infrared emitting fluorochrome analyzable by a scanner. This methodology was first validated by checking that the fluorescent intensity measured with the scanner was well correlated to the measurement done on images obtained by microscopy. Correlations were high, comprising between 0.87 and 0.98 according to the marker. Even if this methodology was less sensitive on weak fluorescent signals than microscopy (see Figures 3, 4 for fibronectin), it allowed us to similarly rank FG muscles according to the occurrence of the WS and WB defects, i.e., for the three markers FG-C < FG-WS < FG-WB < FG-WSWB. However, the low sensitivity of the scanner method makes it difficult to compare SG and FG muscles, certainly because of the very different fiber size likely to bias the signal analysis. This was the case for fibronectin, which appeared about three times less expressed in SG than FG-C muscles when analyzed by microscopy (Figure 3), while no significant difference was observed using the scanning technology (Figure 4). This was also observed for MYH15 (Figure 4). Therefore, we conclude that measuring fluorescence intensity using a scanner on muscle cross-sections may be useful to quickly and precisely diagnosing injuries involved in the occurrence of both WS and WB defects within a population of broilers exhibiting similar growth rate. In addition, the scan methodology used markers involved in fibrosis and muscle fiber regeneration, which are two processes involved in both WS and WB defects. It would be interesting to identify specific markers either of the WS or of the WB, which are compatible with the use of a scanner. Indeed, we have shown that Bodipy 493 is a very good marker of adiposis (Figure 3) and therefore of WS condition, but unfortunately incompatible with the use of our scan technology. Such development will widen the possibilities of fast, specific, and reproducible muscle phenotyping in the future. In the meantime, the histological phenotyping of the muscles can be useful to refine a first visual or spectral phenotyping that easily identifies the presence of WS or WB.

### Which Molecular Processes Differentiate or Are Common to WS and WB Conditions?

By combining histological, expressional, and enzymatic approaches, our study aimed to contribute improving knowledge on the etiology of both WS and WB defects. To achieve this goal, we compared muscle groups that were free from WS and WB or affected by one or the other or both at the same time.

#### Pathways Involved in Muscle Metabolism and Contractile Function

As discussed above, adiposis is a good marker of WS and Bodipy 493 a good histological marker to quantify its occurrence and severity. Among genes we measured, only *FABP4* and *CD36* expression tended to be higher in muscles affected by WS alone or associated with WB. Upregulation of *FABP4* and *CD36* genes was already observed by Papah et al. (2018) and Lake et al. (2019) during the early steps of WB development. Their results suggested that dysregulation of *FABP4*, *CD36*, and *LPL* are likely to be associated with fat deposition in muscle. *FABP4* encodes a fatty acid-binding protein involved in lipid transport in adipocytes. *CD36* codes for the cluster of differentiation 36, a cell surface protein that imports fatty acids inside cells. CD36 binds many ligands including collagen, lipoprotein, phospholipids, and long-chain fatty acids. In relation with their respective function, it is likely that these two proteins participate to recruitment of lipids by adipocytes during their differentiation (Arrighi et al., 2015). *PLIN2* coding an adipose differentiation related protein (perilipin-2) was first characterized as an mRNA molecule that expresses early in adipocyte differentiation (Jiang and Serrero, 1992). *FASN* codes a protein implied in long-chain saturated fatty acids synthesis. Their greater expression in FG compared to SG muscles suggests perilipin-2 and FASN may contribute to the higher adiposis observed in all FG muscles, including normal ones (Figure 3). We also measured the expression of *ADIPOQ* gene coding adiponectin that enhances glucose utilization and fatty-acid combustion. Its expression was not altered by the presence of the defects, which is not in favor of a potential role in their establishment. However, recent results showed that adiponectin content may change following posttranscriptional regulation (Van Pelt et al., 2020).

Among the genes tested, several were specifically regulated in muscles affected with WB compared to normal ones (Table 2). Four of them were downregulated in the presence of WB and more expressed in SG than in FG muscles. They include *MYH1E*, which codes the main form of myosin expressed in adult chicken fast-twitching breast muscle (Bader et al., 1982), and *MYH13*, coding the superfast-twitching, myosin mediating the high-velocity and low-tension contractions of specific striated muscles (Briggs and Schatchat, 2002). On the contrary, *MYH15* coding a slow-twitch ventricular-like myosin heavy chain expressed during regeneration of avian skeletal muscles (Camoretti-Mercado et al., 1993) was significantly upregulated in WB muscles, compared to FG-C; while it was almost not expressed in SG muscles (Figure 8A), as already observed in muscles affected by both WS and WB (Pampouille et al., 2019). Consistently, our study revealed an increased MYH6 signal that it is specific to a slow developmental myosin isoform derived from PM muscle (Miller et al., 1985) in both WS and WB muscles (not shown), arguing in favor of a shift from fast- to slow-twitching as already suggested (Papah and Abasht, 2019). However, the mature form of slow myosin heavy chain (MYH7B, Miller et al., 1985) was not expressed in presence of WS or WB defects (not shown), suggesting that contractile shift induced is mainly due to the replacement of fast IIb toward fast IIa fibers, as observed usually in muscular dystrophies on predominant IIb muscles (Barnard et al., 1982; Pette and Staron, 2001). As revealed by NADH-TR reaction, muscle fiber shift was associated with increased mitochondria density in both WS and WB conditions compared to FG-C muscles (Figure 2). Surprisingly, the increased mitochondria density observed in affected muscle was not accompanied by increased mitochondria COX activity (Figure 5). It was significantly lower in FG than in SG muscles, but not affected by the presence of the defects. in contrast, we observed greater production of ROS in FG than in SG muscles (Figure 5). Altogether, these observations suggest impaired mitochondrial functions in fast-growing muscles, even not affected by WS or WB defects, compared to slow-growing muscles. Another muscle-specific gene, *MYOCD*, was less expressed in FG muscles, especially in the case of WB. It codes myocardin, which is expressed in cardiac muscle and tissues containing smooth muscle cells, in which it may play a crucial role in cardiogenesis and smooth muscle differentiation (van Tuyn et al., 2005). Myocardin has been recently proposed to play a role in the vascularization reduction in the case of severe defects (Pampouille et al., 2019). The last downregulated gene is *ANKRD46* whose function is not known, particularly in connection with myopathies. However, other ankyrins are known to be involved in the costamere, the structure that is responsible for linking sarcomere to the sarcolemma (Maiwailidan et al., 2011). The huge difference in expression (about 125 times; Table 2) that exists between the SG and FG muscles raises questions about its function in chicken muscle and will require further study.

An interesting point is the pattern of expression of *MTX3* that is more expressed in SG than in FG muscles, except in very severely affected FG-WSWB muscles (Figure 9). *MTX3* encodes metaxin 3, which is involved in the protein transport into the mitochondrion. It contains glutathione S-transferase domain (Adolph, 2004) and may therefore participate to detoxification of reactive electrophile species (RES). RES stimulate the expression of cell survival genes, as well as many other genes commonly upregulated in case of stress and pathogenesis. There is evidence that, although excess RES production can lead to cell damage, lower levels of RES may modulate the expression of cell survival genes and by this way contribute to survival during severe stress (for review, see Farmer and Davoine, 2007). Therefore, the upregulation of *MTX3* may contribute to cell survival in chicken muscles severely affected by WS and WB, in coordination with other the pathways such as oxygen transport, axon guidance, and neuromuscular junction repair already reported in these types of muscle (Pampouille et al., 2019).

#### Pathways Involved in Cell Regeneration and Extracellular Matrix Development

Of the genes overexpressed in affected muscles, some were only upregulated when WB was diagnosed. They are mainly involved in connective tissue deposition. *FN1* codes fibronectin, which plays a key role in the adhesion of cells to the ECM. *FN1* expression followed a similar profile as fibronectin histological quantification, suggesting transcriptional regulation (Table 2 and Figure 3). Fibronectin was described as a seric biomarker of Duchenne muscular dystrophy (DMD) (Cynthia Martin et al., 2014), and its expression during fibrosis has been shown to be regulated by *TGFB1* and *CTGF* (Vial et al., 2008), whose expression patterns follow those of *FN1* in our study (Table 2 and Figure 8). *FN1* has been recently proposed as upstream regulator involved in ECM remodeling, which begins at 3 weeks in chickens affected by Wooden Breast (Papah et al., 2018). By contrast, the expression of *FBN1* that codes another ECM glycoprotein fibrillin was not altered by muscle growth or WS or WB condition.

Among the gene-coding proteins of the ECM, some were only upregulated in muscles severely affected by both WS and WB. This is the case of *COL6A3* and *ANTXR1* (Figure 7 and Table 2), whose expression positively correlated (*R* of 0.68; *P* ≤ 0.001). *ANTXR1* encodes a type I transmembrane protein that interacts with collagen types 1 and 6 alpha 3 (coded by *COL6A3*) and may have a role in ECM homeostasis (Hotchkiss et al., 2005). *PDGFRA* encodes platelet-derived growth factor receptor A, also termed PDGFRα, and is described as a marker of fibro-adipogenic progenitors (FAPs), which are required for proper skeletal muscle development, regeneration, and maintenance (Uezumi et al., 2011). However, FAPs are also responsible for fibro-fatty scar deposition following chronic damage (Arrighi et al., 2015; Contreras et al., 2016). In cell culture, PDFGRα positive cells treated by TGFβ1 expressed fibrosis marker like collagen and CTGF contrary to myoblasts (Uezumi et al., 2011). Recently, it was shown that TGFβ inhibits PDFGRα expression in FAPs and promotes myofibroblast differentiation of FAPs but inhibit their adipogenicity (Contreras et al., 2019). The concomitant overexpression of TGFβ, CTGF, and PDFGRα especially in WB muscles argue for their role in controlling FAPs differentiation toward fibroblasts or adipocytes.

Our histological observation brought evidence of cell regeneration with the expression of MYH15 and NCAM proteins in many fibers of muscles affected by WB (Figure 2). We also reported increased expression of several genes involved in myogenesis or cell proliferation in WB muscles. This is the case of *MYOG*, involved in myogenesis and skeletal muscle repair (Figure 7), and of ENPP2 (Figure 8), whose expression increased during myogenic differentiation of C2C12 murine myoblasts and inhibition prevents the differentiation of myoblasts (Sah et al., 2020). In our study, *ENPP2* expression was positively correlated to that of *MYH15* (*R* of 0.70; *P* ≤ 0.001) and of *Twist1* (*R* of 0.72; *P* ≤ 0.001). *Twist1* gene inhibits muscle differentiation by repressing muscle-specific gene activation (Hebrok et al., 1994), which suggests antagonist process either activating or repressing muscle differentiation in WB muscle. However, as described recently, Twist1 has a key role in maintenance of skeletal muscle progenitors (Choi et al., 2020), but also in adipogenesis (Ren et al., 2016). The overexpression of *Twist1* in chicken muscle affected by WB is also consistent with the fact that WB condition has been often associated with a lack of glycogen (Abasht et al., 2016). Indeed, overexpression of *Twist1* and *Twist2* genes reduced total glycogen content in mouse muscle without altering glucose uptake (Mudry et al., 2015). Overexpression of these two genes also increases gene expression of inflammatory cytokines like *IL6*, *TNF*α, and *IL1*β (Mudry et al., 2015). However, in the literature, expression of inflammatory cytokines seems to show some discrepancies, as *IL1*β is downregulated in WB muscle (Zambonelli et al., 2016) while *IL2RG* and *TNFRSF1A* are upregulated (Papah et al., 2018). Further experiments are needed to understand Twist1 role in WB pathogenesis. Increased mitotic activity was also recently reported in muscle affected by WB from 25-day-old chicken, indicating that a regeneration process was underway (Meloche et al., 2018). Accordingly, we reported an overexpression of *PCNA*, specifically expressed in proliferating cells, confirming that cell regeneration was associated with these defects (Figure 9). Interestingly, *PCNA* was also highly expressed in SG muscles, probably because they are at an earlier physiological stage of development than fast-growing muscles. Obviously, regeneration involved in WB disease did not affect *MYF5* and *MYOD* mRNA expressions, the main myogenic factors implied in myogenesis (not shown). Altogether, our observations highlighted several robust markers of the WB condition, most of them being associated with molecular pathways involved in cell proliferation and muscle differentiation and repair following chronic damage. We also pointed out possible antagonist pathways that are either inhibiting or activating myogenic differentiation, whose balance possibly determines muscle progenitor cell fate.

Among genes involved in lipid metabolism, *PPARG* and *FASN* were only significantly upregulated in muscles severely affected by both WS and WB. PPARγ is known as a regulator of adipocyte differentiation, and its expression is inhibited by TGFβ1 in FAPs (Contreras et al., 2019). To evaluate the relevance of the balance between TGFβ1 and PPARγ signaling, we calculated the ratio of *TGFB1* on *PPARG* expression. This ratio was significantly modified depending on the group studied (*P* ≤ 0.01) and was the lowest in SG and the highest in FG-WB muscles (Table 2), suggesting that a high ratio *TGFB1*/*PPARG* would favor the development of a fibrotic rather than adipogenic phenotype. This hypothesis is strengthened because, among FG muscles, lower ratios were observed in muscles exhibiting WS (Table 2), and expression of *PDE3B*, a well-known target of PPARγ (Resnyk et al., 2013; Jahansouz et al., 2018), increased when the *TGFB1*/*PPARG* ratio decreased (*R* of −0.60; *P* ≤ 0.001). Up-regulation of *FASN* and *PDE3B* in a chicken model with higher visceral adiposity supported their potential role on regulation of muscle adiposity (Resnyk et al., 2013).

#### Pathways and Genes Involved in Human Dystrophy

Several genes selected for mRNA expression studies are known to be causative of muscular dystrophies in human. Among them, *DYSF*, *SGCB*, and *COL6A3* are also included in QTL regions controlling WS in chicken breast muscle (Pampouille et al., 2018). In context of mammalian muscular dystrophies involving *COL6A3* mutations, its expression is usually deficient (Pan et al., 2013). In our study, *COL6A3* was highly expressed in fast-growing muscles affected by both WS and WB, probably because of the large amount of fibrosis observed in these muscles (Figure 2). Similarly, *CAV3*, coding caveolin 3, is at least two-times more expressed in FG than in SG muscles. Previously, a 3- to 5-fold increase in caveolin 3 induced an increased vesicular trafficking that was associated with cell necrosis and regeneration and decreased expression of sarcolemmal and subsarcolemmal proteins (Galbiati et al., 2000). However, in the present study, muscle groups did not affect genes coding sarcolemmal proteins SGBC and DAG1 (not shown). Only expression of *CAPN3* was lower in FG compared to SG muscles. *CAPN3* deficiency is associated with limb-girdle muscular dystrophy (Fardeau et al., 1996; Fakhfakh et al., 2012), and its expression is also reduced in degenerating–regenerating mice model and during regeneration process (Stockholm et al., 2001). Its reduced expression in fast-growing compared to slow-growing muscles is therefore consistent with their greater number of degenerating and regenerating cells (Figures 1, 2). Interestingly, *CAPN3* expression is highly correlated (*R* = 0.93; *P* ≤ 0.001) with that of *PPP1R3A*, a major gene controlling the glycogen content in chicken muscles between the high- and the low-pHu line (Le Bihan-Duval et al., 2018). The decrease of its expression in the high-pHu line is associated with lower glycogen content and higher susceptibility to WS defect. Disruption of the *PPP1R3A* gene in mice, encoding the glycogen targeting subunit of protein phosphatase 1, causes substantial lowering of the glycogen synthase activity and decrease in the glycogen levels in skeletal muscle (Delibegovic et al., 2003). It also induces impairment of glucose tolerance and insulin resistance in skeletal muscle associated with increased weight gain and massive abdominal and other fat depositions, likely as a consequence of impaired blood glucose utilization in skeletal muscle. It is therefore suggested that dysfunction of the protein coded by *PPP1R3A* may contribute to the pathophysiology of human type 2 diabetes. This can be related to recent findings in chicken that suggested etiologic similarities between Wooden Breast and type 2 diabetes despite, phenotypic disparities between birds and mammals, likely due to major difference in skeletal muscle glucose transport (Lake and Abasht, 2020). Despite many phenotypic similarities, there is no obvious evidence from our results of common etiology between mammalian dystrophies and myopathies observed in chicken breast muscle. However, genetic and protein studies are underway to explore whether mutations or posttranslational regulations may be involved in chicken myopathies, independently of transcriptional regulations.

## Conclusion

Based on a multi-level biological approach, the present study provided new knowledge and molecular tools useful to characterize WS and WB defects in chicken muscles. Among the most interesting molecular markers identified are those linked to muscle contractile properties and fiber regeneration or involved in the development of fibrosis and adiposis. Our study also highlighted several regulatory pathways that could be decisive for the myogenic, adipogenic, or fibrogenic fate of muscle precursors and key molecules controlling both muscle glycogen and fiber cell regeneration that would deserve further study. In order to improve research on avian myopathies, our study offers the scientific community new rapid histological analysis techniques, based on the use of robust and specific markers, capable of precisely quantifying the injuries induced in the event of WS and WB. Such measurements will allow for a fine assessment the impact of rearing or selection strategies on chicken breast muscle myopathies, but may also serve to provide reliable phenotypes for the future development of non-invasive phenotyping tools based on non-destructive methodologies that can be used on live animals.

## Data Availability Statement

The raw data supporting the conclusions of this article will be made available by the authors, without undue reservation.

## Ethics Statement

The animal study was reviewed and approved by the Ethical Committee for Animal Experimentation in Val de Loire (C2EA-19).

## Author Contributions

CP and CB supervised the study, defined the experimental design and supervised the animal phenotyping with EL and EP. JJ, EG, and NC were in charge of the laboratory analysis under the supervision of CP. CP performed the statistical analyses and drafted the first version of the manuscript under the supervision of CB. All the authors read and approved the final manuscript.

## Conflict of Interest

The authors declare that the research was conducted in the absence of any commercial or financial relationships that could be construed as a potential conflict of interest.

## Supplementary Material

FILE S1 Pearson’s correlations between the relative mRNA levels of genes differentially expressed between *pectoralis major* muscles from a slow-growing genotype (SG) and a fast-growing genotype macroscopically free of defects (FG-C) or affected by White Striping (FG-WS), Wooden Breast (FG-WB), or both White Striping and Wooden Breast (FG-WSWB). Differentially expressed genes are regrouped by cluster (C1–C6). Absolute mRNA levels of targeted genes were corrected for *18S ribosomal RNA* levels to give a relative mRNA level. Pearson’s correlation is indicated when statistically significant (*P* ≤ 0.05) or crossed out with a black cross if it is not.
