# Supplementary figures and images for: Molecular Phenotyping of White Striping and Wooden Breast Myopathies in Chicken
Source: Front Physiol. 2020 Jun 24;11:633. doi: 10.3389/fphys.2020.00633 (PMC7328665; doi:10.3389/fphys.2020.00633)

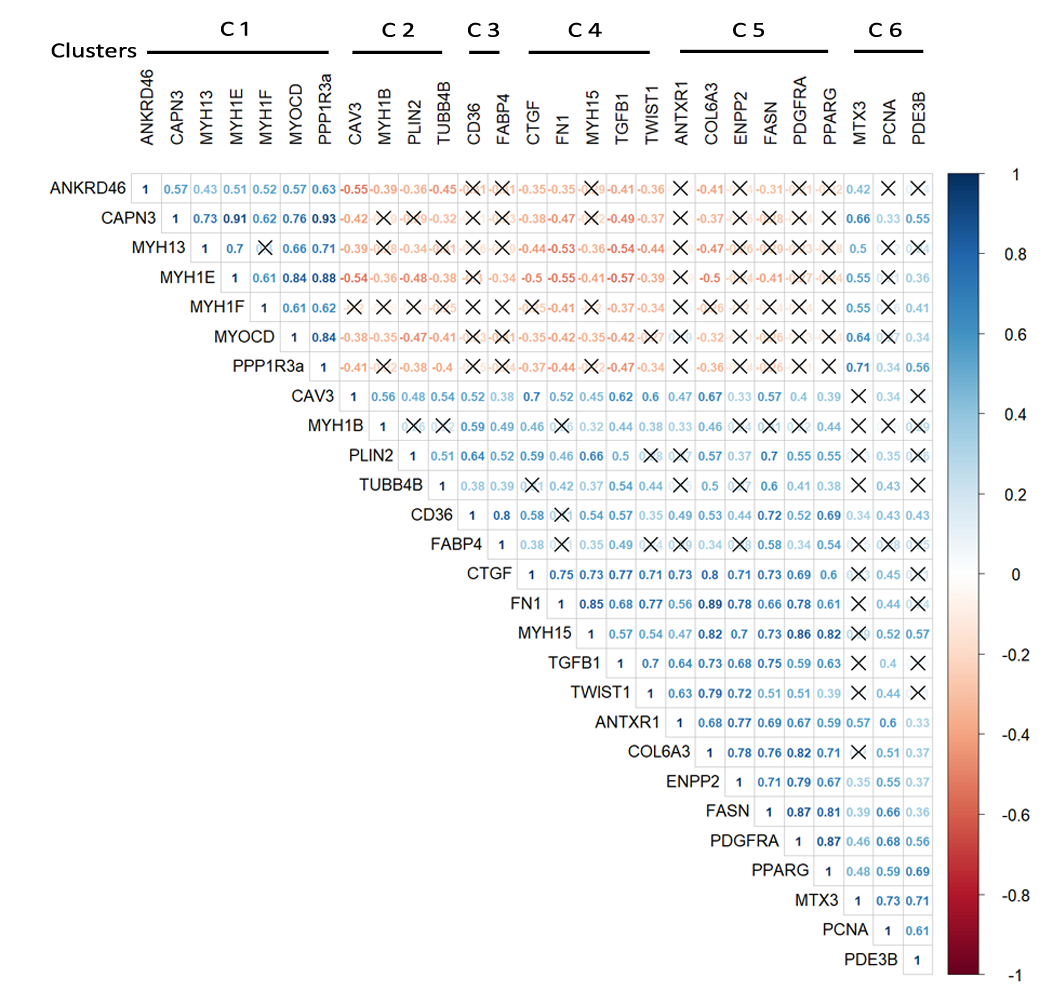

Supplement: FILE S1 — Pearson’s correlations between the relative mRNA levels of genes differentially expressed between pectoralis major muscles from a slow-growing genotype (SG) and a fast-growing genotype macroscopically free of defects (FG-C) or affected by White Striping (FG-WS), Wooden Breast (FG-WB), or both White Striping and Wooden Breast (FG-WSWB). Differentially expressed genes are regrouped by cluster (C1–C6). Absolute mRNA levels of targeted genes were corrected for 18S ribosomal RNA levels to give a relative mRNA level. Pearson’s correlation is indicated when statistically significant (P ≤ 0.05) or crossed out with a black cross if it is not. [file Image_1.TIF]
